# Supplementary material for: Saxiphilin functions as a toxin sponge protein that counteracts the effects of saxitoxin poisoning
Source: Nat Commun. 2026 Jul 16;17:5934. doi: 10.1038/s41467-026-75136-z (PMC13376186; doi:10.1038/s41467-026-75136-z)
Supplement: Supplementary file 1 — Supplementary Information [file 41467_2026_75136_MOESM1_ESM.pdf]

Supplementary Information

Saxiphilin functions as a toxin sponge protein that counteracts the effects of saxitoxin poisoning

Samantha A. Nixon[1#‡], Sandra Zakrzewska[1#], Seil Jang[1], Keli Huang[1], Anissa Bara[2], Zhou Chen[1†], Daynen R. Goss[3], Elizabeth R. Park[3], J. Du Bois[3], and Daniel L. Minor, Jr.[1, 4-8*]

[1]Cardiovascular Research Institute, University of California, San Francisco, CA
[2]Sophion Bioscience, Ballerup, Denmark
[3]Department of Chemistry, Stanford University, Stanford, CA 94305 USA
[4]Department of Biochemistry and Biophysics
[5]Department of Cellular and Molecular Pharmacology
[6]California Institute for Quantitative Biomedical Research
[7]Kavli Institute for Fundamental Neuroscience
University of California, San Francisco, CA 94158-9001 USA

[8]Molecular Biophysics and Integrated Bio-imaging Division
Lawrence Berkeley National Laboratory, Berkeley, CA 94720 USA

[#]Equal contribution

*Correspondence to: daniel.minor@ucsf.edu

[‡] Present address:
Institute for Molecular Bioscience
The University of Queensland
St Lucia, QLD, Australia, 4067

[†]Present address:
Department of Anatomy and Physiology
Shanghai Jiao Tong University School of Medicine
Shanghai, 200025, China

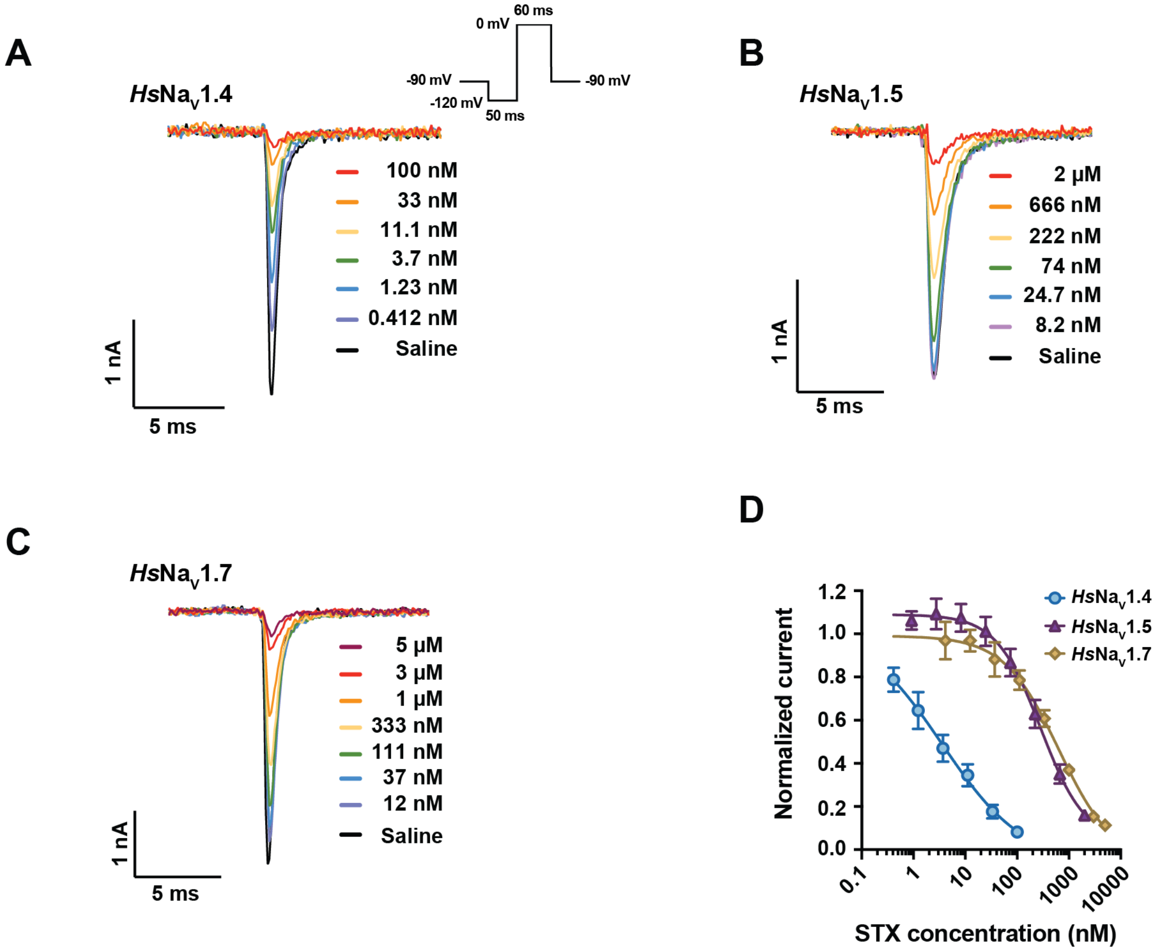

Figure S1 Concentration-response curves for STX against human NaVs. A-C, Exemplar whole cell patch-clamp recordings of increasing concentrations of STX for **A,** *Hs*NaV1.4, **B,** *Hs*NaV1.5, and **C,** *Hs*NaV1.7. **D,** STX-response curves for *Hs*NaV1.4 (blue, circle, $n = 10$), *Hs*NaV1.5 (purple, triangle, $n = 9$), and *Hs*NaV1.7 (gold, diamond, $n = 6$). All points represent mean with SD.

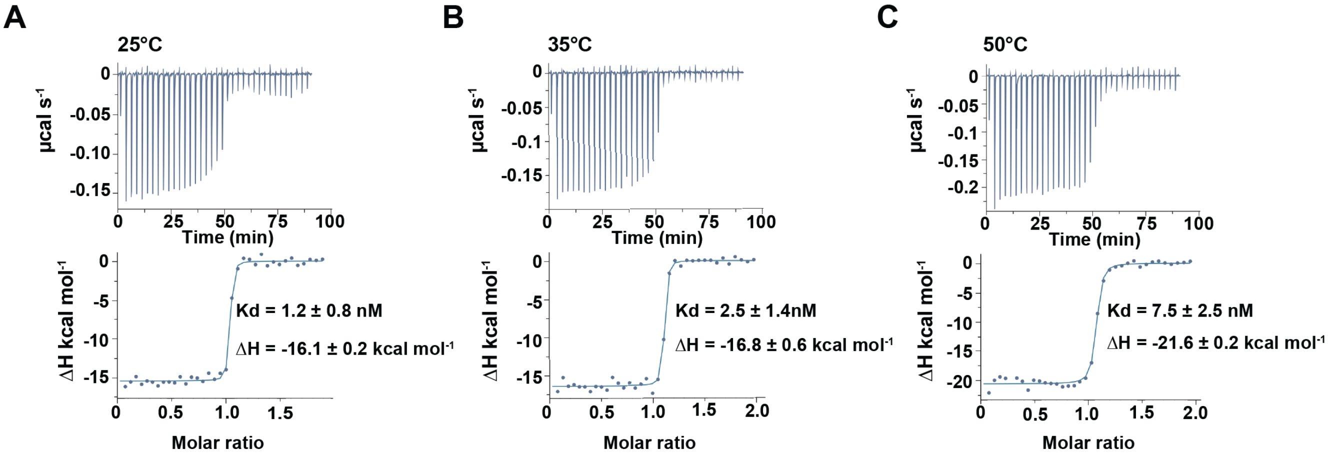

Figure S2 ITC studies for *Rc*Sxph and STX at high temperatures. A-C, Exemplar ITC isotherms for the titration of 100 µM STX into 10 µM *Rc*Sxph at **A,** 25°C (from [1]), **B,** 35°C, and **C,** 50°C**.** Kd and ΔH values are indicated.

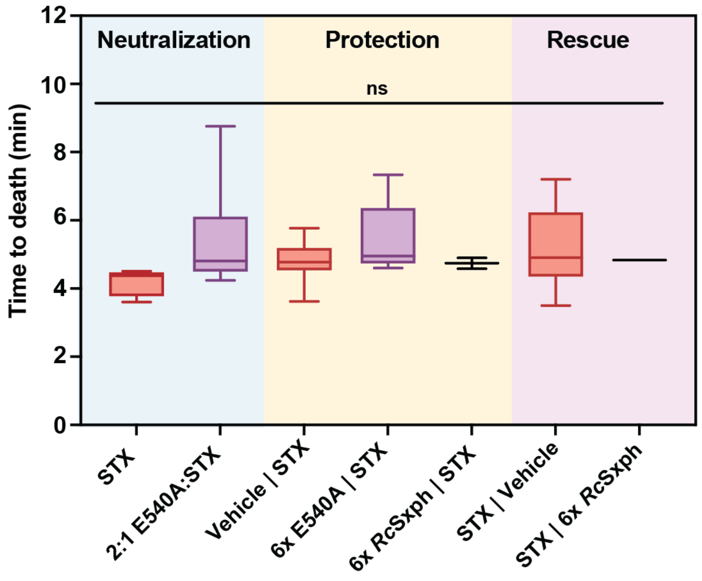

Figure S3 Time to death for STX treated mice in neutralization, protection, and rescue paradigms. Box plots showing the median, minimum, and maximum time to death (minutes) for each treatment group. The doses and number of fatalities in each treatment group were: Neutralization, STX alone (60 nmol/kg, $n = 12$), 2:1 E540A:STX (120 nmol/kg | 60 nmol/kg, $n = 8$); Protection, Vehicle | STX (60 nmol/kg, $n = 11$); 6× E540A |STX (360 nmol/kg | 60 nmol/kg, $n = 9$); 6× *Rc*Sxph | STX (360 nmol/kg | 60 nmol/kg, $n = 2$); Rescue, STX|PBS (60 nmol/kg, $n = 8$), STX|6× *Rc*Sxph (60 nmol/kg | 360 nmol/kg, $n = 1$). Statistical differences between median time of death were compared using Kruskal-Wallis test with Dunn's multiple comparison test, no significant difference between groups was found ($p > 0.05$).

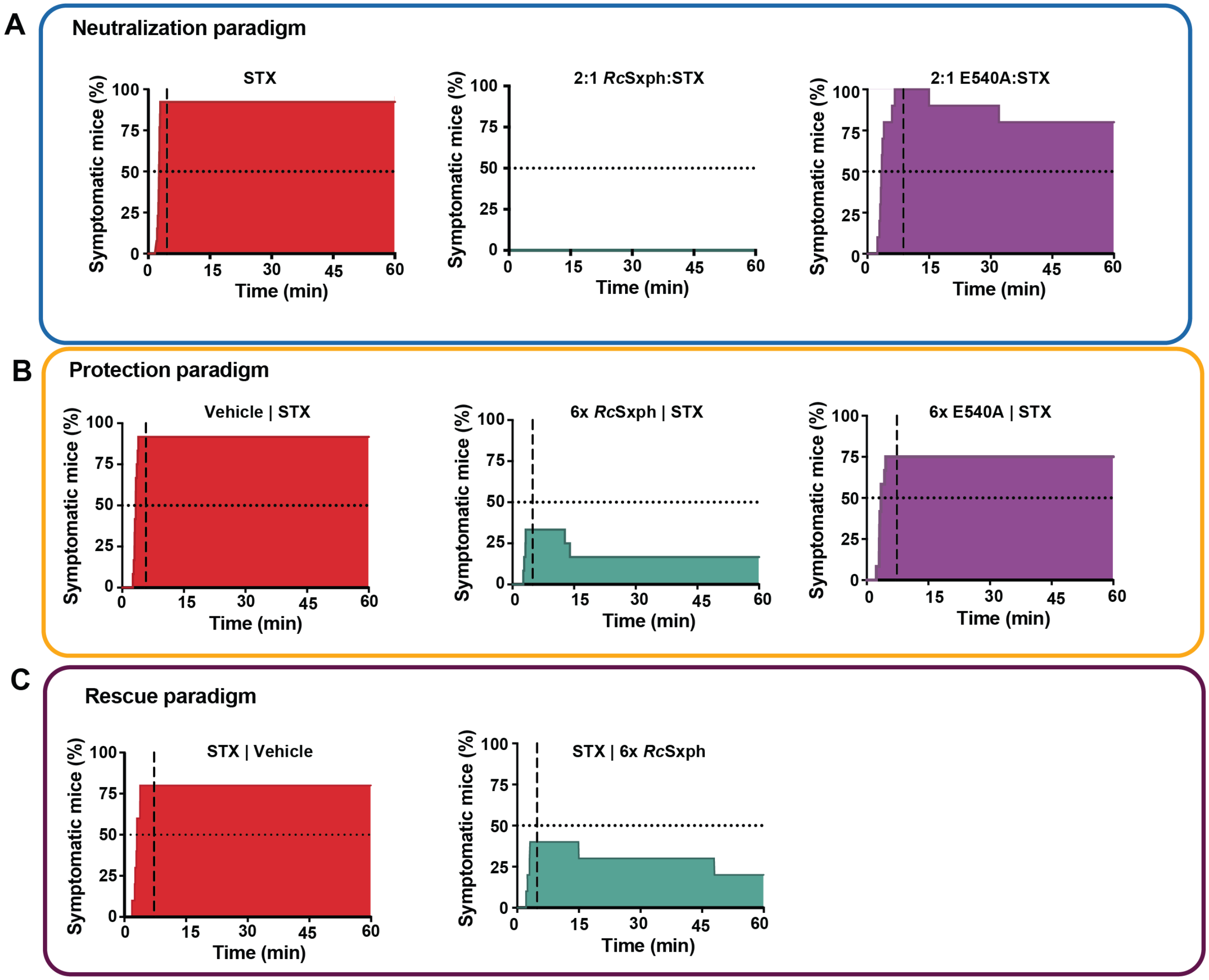

Figure S4 Impact of Sxph treatment on STX poisoning symptoms and recovery over time. Mice displaying STX poisoning symptoms, counted from initial symptom onset (min) were plotted as a percentage of the total treatment group for **A,** *neutralization*, **B,** *protection* and **C,** *rescue* scenarios. The vertical grey dashed line represents the median TOD for each group. Mice were counted as recovered when they had displayed no symptoms for at least 2 minutes and did not develop any further symptoms during the observation period. Mice that died were counted as symptomatic.

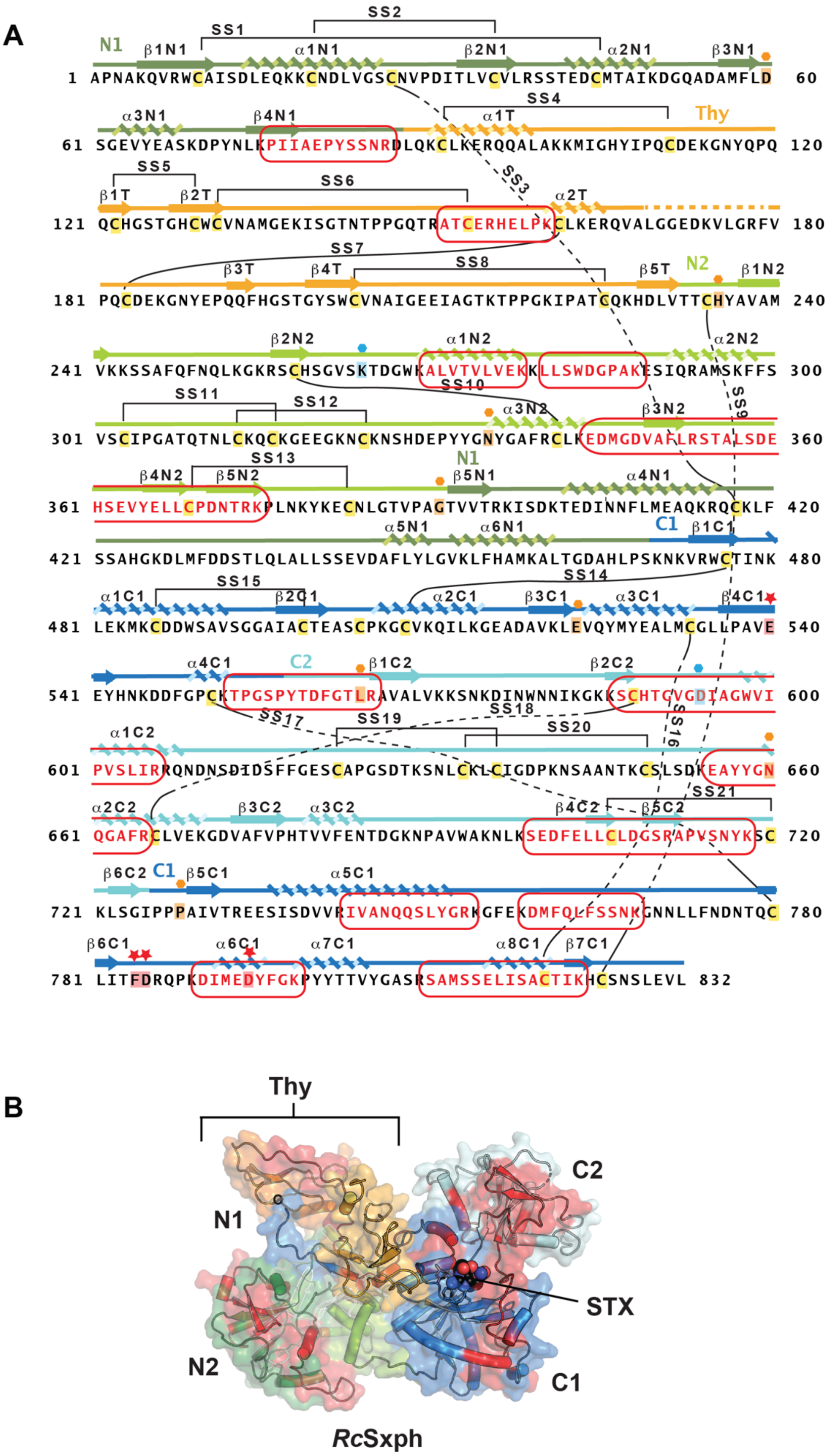
A
N1
β1N1 SS1 α1N1 SS2 β2N1 α2N1 β3N1
1 APNAKQVRWCAISDLEQKKCNDLVGSCNVPDITLVCVLRSSTEDCMTAIKDGQADAMFLD 60
α3N1 β4N1 α1T SS4 Thy
61 SGEVYEASKDPYNLKPIIAEPYSSNRDLQKCLKERQQALAKKMIGHYIPQCDEKGNYQPQ 120
SS5 β1T β2T SS6 SS3 α2T
121 QCHGSTGHCWCVNAMGEKISGTNTPPGQTRATCERHELPKCLKERQVALGGEDKVLGRFV 180
SS7 β3T β4T SS8 β5T N2 β1N2
181 PQCDEKGNYEPQQFHGSTGYSWCVNAIGEEIAGTKTPPGKIPATCQKHDLVTTCHYAVAM 240
β2N2 α1N2 α2N2
241 VKKSSAFQFNQLKGKRSCHSGVSKTDGWKALVTVLVEKKLLSWDGPAKESIQRAMSKFFS 300
SS11 SS12 SS10 α3N2 β3N2 SS9
301 VSCIPGATQTNLCKQCKGEEGKNCKNSHDEPYYGNYGAFRCLKEDMGDVAFLRSTALSDE 360
β4N2 β5N2 SS13 N1 β5N1 α4N1
361 HSEVYELLCPDNTRKPLNKYKECNLGTVPAGTVVTRKISDKTEDINNFLMEAQKRQCKLF 420
α5N1 α6N1 C1 β1C1
421 SSAHGKDLMFDDSTLQLALLSSEVDAFLYLGVKLFHAMKALTGDAHLPSKNKVRWCTINK 480
α1C1 SS15 β2C1 α2C1 SS14 β3C1 α3C1 β4C1
481 LEKMKCDDWSAVSGGAIACTEASCPKGCVKQILKGEADAVKLEVQYMYEALMCGLLPAVE 540
α4C1 C2 β1C2 β2C2
541 EYHNKDDFGPCKTPGSPYTDFGTLRAVALVKKSNKDINWNNIKGKKSCHTCVGDIAGWVI 600
SS17 SS19 SS18 SS16 α1C2 SS20
601 PVSLIRRQNDNSDIDSFFGESCAPGSDTKSNLCKLCIGDPKNSAANTKCSLSDKEAYYGN 660
α2C2 β3C2 α3C2 β4C2 β5C2 SS21
661 QGAFRCLVEKGDVAFVPHTVVFENTDGKNPAVWAKNLKSEDFELLCLDGSRAPVSNYKSC 720
β6C2 C1 β5C1 α5C1
721 KLSGIPPPAIVTREESISDVVRIVANQQSLYGRKGFEKDMFQLFSSNKGNNLLFNDNTQC 780
β6C1 α6C1 α7C1 α8C1 β7C1
781 LITFDRQPKDIMEDYFGKPYYTTVYGASRSAMSSELISACTIKHCSNSLEVL 832
B
Thy
N1
C2
STX
N2
C1
*Rc*Sxph

Figure S5 Map of observed *Rc*Sxph peptide fragments. A, Shared observed peptides from mouse organs following 5-hour *Rc*Sxph treatment. **B,** Location of peptides from 'A' (red) mapped on the *Rc*Sxph:STX structure (PDB: 6O0F)[2]. Domains are indicated and are colored as follows: N1 (smudge), N2 (limon), thyroglobulin (Thy; bright orange), C1 (marine), and C2 (cyan). STX (black) is shown as space filling.

Table S1 STX IC_{50}s for *Hs*Na_V1.4, *Hs*Na_V1.5 and *Hs*Na_V1.7 and STX and Sxph concentrations used for *in vitro* neutralization studies

Isoform	IC_{50} (nM)	*n*	[STX] [Sxph] *in vitro*	
			STX (nM)	Sxph (nM)
***Hs*Na_V1.4**	3.5 ± 2.5	10	100	300
***Hs*Na_V1.5**	284.6 ± 50.[illegible]	9	1100	3300
***Hs*Na_V1.7**	637.9 ± 189	6	3000	9000

IC_{50}, half-maximal inhibitory concentration

n, number of cells

Errors are SD.

Table S2 *Rc*Sxph:STX thermodynamic binding parameters

Temperature	N (sites)	Kd (nM)	ΔH (kcal mol^{-1})	ΔS (cal mol^{-1} K^{-1})	ΔG (kcal mol^{-1})	ΔΔG (kcal mol^{-1})	n
**25°C***	1.02 ± 0.01	1.2 ± 0.8	-16.1 ± 0.2	-12.7 ± 0.9	-12.3 ± 0.5	-	3
**35°C**	1.02 ± 0.01	2.5 ± 1.4	-16.8 ± 0.6	-15.1 ± 3.3	-12.2 ± 0.4	0.1	2
**50°C**	1.01 ± 0.06	7.5 ± 2.5	-21.6 ± 0.2	-29.7 ± 0.6	-12.0 ± 0.0	0.3	2

N, number of binding sites

Kd, dissociation constant

$\Delta\Delta G = \Delta G_{T(°C)} - \Delta G_{25°C}$

n, number of observations

* Data taken from [1]

Errors are SD.

Table S3 Median time of death (TOD) for STX treated mice in neutralization, protection, and rescue paradigms

Paradigm	Treatment group	Median TOD (min)	95% CI	*n*
**Neutralization**	STX alone	4.38	3.75–4.5	12
	2:1 E540A:STX	4.8	4.23–8.75	8
**Protection**	Vehicle | STX	4.77	4.47–5.26	11
	6x E540A | STX	4.95	4.68–7.0	9
	6x *Rc*Sxph | STX	4.74	4.58–4.9	2
**Rescue**	STX | Vehicle	4.91	3.5–7.2	8
	STX | 6x *Rc*Sxph	4.83	nd	1

nd = not determined due to insufficient *n*

95% CI, 95% confidence interval

n number of fatalities

Table S4 Behavioral markers and associated seizure severity score

	Seizure Type	Score	Behavioral markers	Onset (min)	STX-treatment observational notes
Increasing seizure severity	**None**	-1	Normal behavior	N/A	
	**Patrial/focal**	0	Whisker trembling	N/A	Whisker trembling was indistinguishable from control mice.
		1	Sudden behavioral arrest	2–3 min	Observed in STX-treated mice. Not counted due to lack of correlative EEG.
		2	Facial jerks	2–3 min	Observed in STX-treated mice. Not counted due to lack of correlative EEG.
	**Generalized**	3	Head and neck jerks	2.5–3.5 min	Observed in STX-treated mice.
		4	Clonic seizure (sitting)	N/A	Not observed in any animals in this study.
		5	Clonic, tonic-clonic seizure with animal on belly (loss of muscle control)	2.5–4 min	Observed.
		6	Clonic, tonic-clonic seizure with animal lying on side or wild jumping	3–4 min	Observed barrel-rolls in score 6 for STX-induced seizures.
	**Death**	7	Tonic extension (laying down with limbs outstretched), death	3–5 min	Tonic extension was accompanied by abdominal breathing, indicating respiratory paralysis.

Scores using modified Racine scale for PTZ-induced seizures in mice [3] with associated time of onset (min) following STX administration (60 nmol/kg i.p.) and observational notes.

Scores 3 and above were counted.

Supplementary Methods

Mass spectrometry

Spray voltage was set to 1600 V and ion transfer tube temperature to 275°C. MS1 scans of peptide precursors were analyzed in Orbitrap in range 145-1450 *m/z* at 60K resolution and with following settings: RF Lens 60%, maximum injection time 123 ms, AGC target 100. DIA scans were performed in Orbitrap at 30K resolution. AGC target was set to 1000 and maximum injection time mode to Auto. Precursor mass range 400-1000 *m/z* was covered by 30 windows 20 Da wide. Activation type was set to HCD with 28% collision energy.

Supplementary References

1 Chen, Z. *et al.* Definition of a saxitoxin (STX) binding code enables discovery and characterization of the anuran saxiphilin family. *Proc Natl Acad Sci U S A* **119**, e2210114119 (2022). https://doi.org:10.1073/pnas.2210114119

2 Yen, T.-J., Lolicato, M., Thomas-Tran, R., Du Bois, J. & Minor, D. L., Jr. Structure of the Saxiphilin:saxitoxin (STX) complex reveals a convergent molecular recognition strategy for paralytic toxins. *Sci Adv* **5** (2019). https://doi.org:10.1126/sciadv.aax2650

3 Van Erum, J., Van Dam, D. & De Deyn, P. P. PTZ-induced seizures in mice require a revised Racine scale. *Epilepsy Behav* **95**, 51-55 (2019). https://doi.org:10.1016/j.yebeh.2019.02.029
