# Supplementary material for: Saxiphilin functions as a toxin sponge protein that counteracts the effects of saxitoxin poisoning
Source: Nat Commun. 2026 Jul 16;17:5934. doi: 10.1038/s41467-026-75136-z (PMC13376186; doi:10.1038/s41467-026-75136-z)
Supplement: Supplementary file 2 — Reporting Summary [file 41467_2026_75136_MOESM2_ESM.pdf]

nature portfolio

Corresponding author(s): Daniel L. Minor, Jr., Ph.D.

Last updated by author(s): May 27, 2026

Reporting Summary

Nature Portfolio wishes to improve the reproducibility of the work that we publish. This form provides structure for consistency and transparency in reporting. For further information on Nature Portfolio policies, see our Editorial Policies and the Editorial Policy Checklist.

Statistics

For all statistical analyses, confirm that the following items are present in the figure legend, table legend, main text, or Methods section.

n/a	Confirmed	
☐	☒	The exact sample size (*n*) for each experimental group/condition, given as a discrete number and unit of measurement
☐	☒	A statement on whether measurements were taken from distinct samples or whether the same sample was measured repeatedly
☐	☒	The statistical test(s) used AND whether they are one- or two-sided<br>*Only common tests should be described solely by name; describe more complex techniques in the Methods section.*
☐	☒	A description of all covariates tested
☐	☒	A description of any assumptions or corrections, such as tests of normality and adjustment for multiple comparisons
☐	☒	A full description of the statistical parameters including central tendency (e.g. means) or other basic estimates (e.g. regression coefficient) AND variation (e.g. standard deviation) or associated estimates of uncertainty (e.g. confidence intervals)
☐	☒	For null hypothesis testing, the test statistic (e.g. *F*, *t*, *r*) with confidence intervals, effect sizes, degrees of freedom and *P* value noted<br>*Give P values as exact values whenever suitable.*
☒	☐	For Bayesian analysis, information on the choice of priors and Markov chain Monte Carlo settings
☒	☐	For hierarchical and complex designs, identification of the appropriate level for tests and full reporting of outcomes
☒	☐	Estimates of effect sizes (e.g. Cohen's *d*, Pearson's *r*), indicating how they were calculated

Our web collection on statistics for biologists contains articles on many of the points above.

Software and code

Policy information about availability of computer code

Data collection	Whole-cell patch clamp data were acquired using Sophion Software (Sophion Bioscience).
Data analysis	Sophion Analyzer Software (Sophion Bioscience)<br>GraphPad Prism 10.0 (GraphPad Software)<br>MicroCal PEAQ-ITC Analysis Software (Malvern Panalytical)<br>Spectronaut 18 (Biognosys), Perseus 1.6.15.0, iMovie v10.4.3 (Apple).

For manuscripts utilizing custom algorithms or software that are central to the research but not yet described in published literature, software must be made available to editors and reviewers. We strongly encourage code deposition in a community repository (e.g. GitHub). See the Nature Portfolio guidelines for submitting code & software for further information.

April 2023

Data

Policy information about availability of data

All manuscripts must include a data availability statement. This statement should provide the following information, where applicable:

- Accession codes, unique identifiers, or web links for publicly available datasets
- A description of any restrictions on data availability
- For clinical datasets or third party data, please ensure that the statement adheres to our policy

Mass spectroscopy data have been deposited at the Mass Spectrometry Interactive Virtual Environment (https://massive.ucsd.edu) under identifier MSV000099884. Data or materials will be provided on request from the corresponding author.

Research involving human participants, their data, or biological material

Policy information about studies with human participants or human data. See also policy information about sex, gender (identity/presentation), and sexual orientation and race, ethnicity and racism.

Reporting on sex and gender	Not applicable
Reporting on race, ethnicity, or other socially relevant groupings	Not applicable
Population characteristics	Not applicable
Recruitment	Not applicable
Ethics oversight	Not applicable

Note that full information on the approval of the study protocol must also be provided in the manuscript.

Field-specific reporting

Please select the one below that is the best fit for your research. If you are not sure, read the appropriate sections before making your selection.

☒ Life sciences ☐ Behavioural & social sciences ☐ Ecological, evolutionary & environmental sciences

For a reference copy of the document with all sections, see nature.com/documents/nr-reporting-summary-flat.pdf

Life sciences study design

All studies must disclose on these points even when the disclosure is negative.

Sample size	For whole-cell patch clamp, the minimum sample size per experimental cell condition was at least six independent cells (n=6). The n value for each experiment is reported in each Figure panel.<br><br>For STX survival experiments in mice, minimum sample sizes per treatment group were determined by using expected survival proportions in power analyses for comparing proportions of two independent populations. Values for significance () and power were set to 0.05 and 0.8 respectively. The survival proportions for STX only (p1) and Sxph intervention (p2) were conservatively approximated to 0.2 and 0.8, respectively, to give a minimum final sample size of n = 10. All power analyses were conducted using the University of British Columbia, Canada web-based calculator (https://www.stat.ubc.ca/~rollin/stats/ssize/b2.html
Data exclusions	Any cells which did not meet minimum parameters (resistance at least 0.1 gOhm, capacitance at least 10 pF, peak current at least 0.5 nA, leak current greater than 0.1 nA), or that did not respond to positive control (application of toxin sufficient to block Na currents) were excluded from the experiment.
Replication	All replication attempts were successful.
Randomization	For the whole-cell patch clamp, the results are from at least six independent cells. n=6-10. ('n' value is shown for each panel).
Blinding	Experiments were not blinded due to environmental health and safety and protocol requirements for STX handling.

Reporting for specific materials, systems and methods

We require information from authors about some types of materials, experimental systems and methods used in many studies. Here, indicate whether each material, system or method listed is relevant to your study. If you are not sure if a list item applies to your research, read the appropriate section before selecting a response.

Materials & experimental systems

n/a	Involved in the study	
x		Antibodies
	x	Eukaryotic cell lines
x		Palaeontology and archaeology
	x	Animals and other organisms
x		Clinical data
x		Dual use research of concern
x		Plants

Methods

n/a	Involved in the study	
x		ChIP-seq
x		Flow cytometry
x		MRI-based neuroimaging

Eukaryotic cell lines

Policy information about cell lines and Sex and Gender in Research

Cell line source(s)	HsNaV1.4 in HEK293 Griptite cells (SB Drug Discovery, SB-HEK-hNaV1.4); HsNaV1.5, in HEK293 cells (SB Drug Discovery SB HEK hNaV1.5); and TE671 cells endogenously expressing HsNaV1.7 (ATCC, RD-CCL-136).
Authentication	Inhibition of NaV peak currents by STX using QPatch II (Sophion Bioscience).
Mycoplasma contamination	All delivered cells are free of mycoplasma.
Commonly misidentified lines (See ICLAC register)	Not applicable

Animals and other research organisms

Policy information about studies involving animals; ARRIVE guidelines recommended for reporting animal research, and Sex and Gender in Research

Laboratory animals	Experiments were conducted using 4–6-week-old female CD-1 IGS mice (17–26.3 g, mean weight at dosing) sourced from Charles River Laboratories, USA.
Wild animals	No wild animals were used in this study.
Reporting on sex	Female mice were used in this study.
Field-collected samples	No field collected samples were used in this study.
Ethics oversight	All animal experiments were approved by the Institutional Animal Care and Use Committee (IACUC), University of California, San Francisco (protocol approval #AN076215) and performed in accordance with University of California guidelines and regulations.

Note that full information on the approval of the study protocol must also be provided in the manuscript.

Plants

Seed stocks	Not applicable
Novel plant genotypes	Not applicable
Authentication	Not applicable
