# Supplementary material for: Saxiphilin functions as a toxin sponge protein that counteracts the effects of saxitoxin poisoning
Source: Nat Commun. 2026 Jul 16;17:5934. doi: 10.1038/s41467-026-75136-z (PMC13376186; doi:10.1038/s41467-026-75136-z)
Supplement: Supplementary file 7 — Transparent Peer Review file [file 41467_2026_75136_MOESM7_ESM.pdf]

nature portfolio

Peer Review File

Saxiphilin functions as a toxin sponge protein that counteracts the effects of saxitoxin poisoning

Corresponding Author: Professor Daniel Minor

This file contains all reviewer reports in order by version, followed by all author rebuttals in order by version.

Version 0:

Reviewer comments:

Reviewer #1

(Remarks to the Author)
STX is a paralytic toxin that inhibits Nav activity, blocking bioelectrical signaling. Given that STX can be deadly and poses a health risk to humans, there is interest in developing an effective antidote. Sxphs are STX binding proteins that provide STX resistance to frogs. In this manuscript, Nixon et al. test the hypothesis that Sxphs act as STX sponges that can reduce neurotoxicity and lethality in mice. Nixon et al. combine in vitro and in vivo experiments to show that Sxphs reduce neurotoxic effects and lethality in mice.

There is a substantial body of literature on STX and Sxphs, and this study builds on previous work by testing “toxin sponge” countermeasure hypotheses using multiple paradigms in a mouse model. The strength of this study lies in the combination of in vitro and in vivo experiments (including three paradigms to assess the efficacy of Sxphs to counteract STX neurotoxicity in mice) showing the capacity of Sxphs to reduce the effects of STX on both Nav targets and mice (both neurotoxic effects and lethality). The proteomic data showing the presence of Sxphs in different organs after i.p. injections is also a strength of the paper. The findings support the idea that Sxphs can be used to develop an antidote to STX. The potential to use Sxphs to develop STX antidotes could have significant impacts on human health and food safety.

The experimental design, data, and presentation are solid, and the statistical tests are appropriate. Overall, the data support the author’s interpretations and conclusions. In addition, the text is written in a clear and concise manner, and the literature is appropriate. There are a few typos that should be revised, otherwise there are no suggestions for substantial revisions.

Reviewer #2

(Remarks to the Author)
Dear Authors,

I had the pleasure of reading and reviewing the submitted article titled: “Saxiphilin functions as a ‘toxin sponge’ protein that counteracts the effects of saxitoxin poisoning”. The authors have an excellent voice and convey the topics with clarity and a depth of understanding that makes the manuscript easy to digest. The article discusses their continued work with “toxin sponges”, primarily Sxph, as a mediator for neurotoxin STX. What is new in this article compared to their past work is primarily the inclusion of animal models susceptible to the neurotoxin’s deadly effects and the protection and recovery that Sxph offers. I will not be commenting on the multitude of very well described technologies such as electrophysiology and will be focusing on the proteomics experimentation and results. While the initial results are promising, there is a lack of detail in the description of the sample preparation, mass spectrometry data acquisition and the mass spectrometry data analysis. Without additional details, the results are unable to be evaluated correctly or with a standard desired by the field. The experiments themselves could be sound, but without more information, making those judgements is challenging. Most noteworthy is the claim that Sxph is present in all harvested organs treated but not in those untreated. More interpretation of the results is required to validate with high confidence. Supplemental table 5, for example, utilizes software-specific headers which would be best described for those either without software access and/or in a vendor-neutral manner. With better header descriptions, the raw data comparisons may make more sense and agree with the authors’ claims. Secondarily, the proteomics results were utilized, though only briefly mentioned, to validate safety. The authors do not mention what parameters were used to evaluate the proteome in this manner. I have broken down my specific thoughts and comments

below and by section so that hopefully the authors can remark on these concerns in a manner this is expedient to them.

Intro
“Electrophysiological experiments have shown that binding of STX and STX congeners to this site endows Sxphs with the ability to rescue NaVs from block by these toxins.”
This sentence reads awkwardly and the narrative would be more easily followed if this were improved.

“…and that the high-affinity of Sxph:STX interaction is stably maintained across a wide temperature range that includes typical mammalian body temperatures.”
Can the authors please explain why the interaction between protein and toxin would be affected by various temperature regimes or provide a reference for the readers to indicate the relationship between temperature and interaction?

Protein digestion
- Part numbers would be appreciated for the reagents used in this protocol, particularly the model number of the BCA assay kit and model numbers for the lysis buffer solutions so those attempting to follow the method can be assured they are selecting the correct purity.
- I do not see the additional of any reagents that would be used for reduction and alkylation of the proteins prior to digestion (typically IAA and DTT. Were these added? Do you anticipate that not modifying these residues would affect your results? If you did reduce and alkylate, please state where in the protocol and how this was performed.
- What manufacturer or kit were you using for the SP3 beads?
- A figure or a more detailed protocol for the steps used by the automated sample handler would be appreciated as it will help the reader follow the steps utilized even without the specialized lab equipment.
- Was the overnight digestion also performed at 40°C?
- Please describe the trypsin used for these experiments by manufacturer, catalogue number, etc. Was the trypsin used MS-grade?

nLC-MS/MS
- While not entirely necessary, providing a part number for the column used would be beneficial to readers or those trying to repeat results.
- Was the column heated? If so, to what temperature?
- Was the trap column heated? If so, to what temperature?
- Manufacturers and grade of solvents used for LC-MS/MS should also be included here, especially if the formic acid was pre-mixed.
- Please include the model of the nano-LC/UHPLC used here.
- A full LC gradient would be appreciated, though I acknowledge that the peptide elution range was 60 minutes from 4 to 35% increasing in %B.
- The peptides were converted to gas phase via nano electrospray ionization, which while still a form of desolvation, should be clarified. Including the model of ion source, considering Thermo’s wide range, would be useful to the reader if attempting to repeat results.
- 4 x 10^5, the 5 should be superscript and or could be written as 4x10E5 for the ion count target.
- Please ensure that m/z is italicized following normal conventions in mass spectrometry.
- Carryover between injections can be a significant problem in LC-MS/MS; please describe the sample injection table including when blanks were injected and what types of blanks were utilized. Was any blank subtraction performed in analysis? While not entirely necessary, ensuring that blanks contain few proteins is crucial.
- What were the injection volumes? How much sample was injected onto column?
- A table of instrument parameters would be useful in the supplemental including what was already provided and additionally spray voltage, ion transfer tube temperature, RF lens percentage or Vpp, injection time values, data type (centroid/profile), and other relevant MS-related parameters.

Proteomic Data Analysis
- Inclusion of the FASTA file into MASSIVE or another repository would be ideal.
- An example of tables and values that are useful for data analysis can be seen here: https://pubs.acs.org/doi/full/10.1021/acs.jproteome.3c00671 this publication often serves as a good baseline for variables utilized within the Spectronaut software to ensure robust results. The authors did include more stringent search values than the default, which is greatly appreciated.
- How were the different organs searched? As a group? Separately? Spectronaut can often infer peptide identifications based on other datasets included in a group, so knowing the grouping can help the reader evaluate uniqueness between organ systems.
- The authors state that they utilize Perseus for downstream analysis but do not provide a table or instructions on how to repeat their results (or even what Perseus was used for).

Results
- Non-endogenous proteins can often undergo modification when introduced into a host system. Did the measured amount of Sxph in the organ systems track how much Sxph was introduced to the animal? Was there an expected loss of intact protein due to modifications?
- How much Sxph was detected in organ system blanks? Theoretically, there should be none, but this would be excellent confirmation of LC-MS/MS related systematic carryover between injections and/or confirmation that the data analysis

pipeline did not introduce any false positives downstream.
- By what definition is a “stress-response” protein? This claim would be better supported by a pathway analysis utilizing the quantitative nature of the data (as described in the methodology).
- A cursory inspection of the proteomics data does yield a concerning result which would be alleviated with a deeper description of the injection organization and search results. Uromodulin, a protein exclusively secreted by the kidney in the thick ascending limb, was present in the liver proteomics supplemental data table. Providing definitions for the headers for each column (for example, does NrOfStrippedSequencesIdentified mean, in layman terms, number of unique tryptic peptides?).
- Is there an experimental reason why the liver and skeletal muscle have ~2000 fewer identified protein groups as the other organs? Did these samples have less on-column load?
- Can the authors comment on how bioavailability in multiple organ systems can be beneficial for a sodium-channel blocking neurotoxin like STX?
- Where in the supplemental table 5 does it indicate that there was no Sxph signal? The binary results are generally some sort of computation related back to either integrated intensity from the chromogram for each ID’ed peptide in the protein group. So, in the kidney data table, row 7326 for Sxph, 40 peptides were able to be mapped across all samples in the group (assuming just kidney) but the vehicle, while in lower quantity, did have measurable signal for this protein. If there is additional computation occurring for this table in which the magnitude of difference between the vehicle and dosed samples shows some statistical significance, please describe.
- Was supplemental figure 5 generated with a combination of all ID’ed peptides for the protein across all organs? Why not just perform LC-MS/MS on the isolated protein to perform this experiment? Was the binding pocket region mapped by LC-MS/MS? That would be an interesting result to highlight, particularly if the pi-interactions are crucial for the binding affinity.

I appreciate the immense work that has gone into this study and hope that my concerns are alleviated with additional experimental details.

Reviewer #3

(Remarks to the Author)
In this article, Nixon and her colleagues reexamine the ability of saxiphilin (Sxph), a protein produced naturally by certain species of frogs, to sequester saxitoxin (STX), a highly potent neurotoxin generated by microalgae that can accumulate in shellfish. By blocking voltage-gated sodium channels, STX triggers severe symptoms within minutes potentially leading to death and requiring urgent medical intervention. Using in vitro tests and mouse models, the authors demonstrate that RcSxph from the American bullfrog effectively neutralizes STX on several subtypes of human sodium channels and functions effectively as a “toxin sponge.” In mice, a single intraperitoneal dose of RcSxph protected against lethal exposure to STX, whether administered before, during, or after toxin exposure. This protection was dependent on an intact STX binding pocket, confirming direct sequestration of the toxin in vivo. RcSxph was shown to rapidly distributed throughout the body, allowing it to capture circulating STX and prevent neurotoxicity. Compared to previous antiserum-based strategies, RcSxph was more effective at higher toxin doses without requiring intravenous administration or encapsulation. The protein was well tolerated, stable, and non-toxic. It should be noted that Sxphs bind to several STX congeners, suggesting broad protection against these toxins. Overall, these results validate the “toxin sponge” hypothesis and highlight Sxphs as promising and scalable antidote candidates for STXs and related neurotoxins.

As with the group's previous work, the experiments are exceptionally well conducted and the results are supported by rigorous and appropriate controls. The stated goal of developing a new strategy to counter saxitoxin poisoning in mammals, as well as its experimental validation, are clearly and convincingly demonstrated. That said, several aspects of the discussion could be further developed to strengthen the broader interpretation of the results. The authors explore three different approaches to Sxph administration—before, during, or after STX injection—representing three distinct paradigms that all result in effective toxin neutralization. While these results highlight the potential of Sxph as a preventive, protective or therapeutic agent, and provide valuable insights into its mechanism of action (particularly with regard to its ability to cross the blood-brain barrier), additional context would help clarify their relevance in real-world scenarios of food poisoning. For example, the practical value of administering an antidote preventively, or even at the time of ingestion, may be limited, since it would generally suffice to avoid consuming contaminated shellfish. Furthermore, as both the toxin and the antidote were administered intraperitoneally, an oral exposure model might better reflect the natural route of poisoning if the aim is to propose a strategy applicable to the ingestion of contaminated food. With this in mind, the results of a previous study, rightly cited in the manuscript, could have been discussed (Finch et al., 2023). It would also be interesting to test sublethal doses of STX and inject RcSxph after the first symptoms appear. Finally, the immunogenicity of RcSxph, a 91 kDa protein, could pose a significant problem in the event of repeated use as an antidote. This point could also be discussed and solutions proposed.

Reviewer #4

(Remarks to the Author)
Using mouse models of STX poisoning, Nixon and co-workers demonstrated that Saxiphilins (Sxphs) have the potential to be an effective anti-STX agent. Indeed, they showed that RcSxph from the American bullfrog (Rana catesbeiana) efficiently counteracts the lethal effects of STX poisoning in vivo, in three different murine toxin challenge paradigms, each of them involving injection in mice of a STX dose known to induce the rapid onset of paralysis, seizures, convulsions, and death within few minutes. RcSxph rapidly distributes in mice organs, including brain, and it appears to be tolerated in mice without

any apparent acute toxicity or adverse reactions, at least for the entire duration of the experiments. I found the work highly compelling in defining Sxphs as a prototypical antidote for the lethal toxin STX, and more in general, for paralytic shellfish toxins, of which STX is the archetype. In this regard, I found it particularly strickling that a single dose of RcSxph counteracts STX toxicity/lethality in mice in either order of STX and Sxph administration (together or sequentially).

Also, the preliminary biochemical characterization of RcSxph stability/binding affinity for STX (i.e., ITC experiments) is clear and thus convincing. I just have a curiosity:

The authors reported that RcSxph has an apparent melting temperature (Tm) of ~55°C. I wonder whether the authors encountered 'heat issue' in the baseline stabilization of ITC measurements performed at 50°C. This is, indeed, a value close to the reported melting temperature of the protein and, therefore, folding and unfolding phenomenon should equally occur in this condition. Did the authors see the occurrence of this thermodynamic folding and unfolding equilibrium, perhaps visualized in a longer time to reach baseline stabilization and/or in a significantly different heat value of the baseline with respect to ITC measurements performed at 25°C and 35°C?

Version 2:

Reviewer comments:

Reviewer #1

(Remarks to the Author)
The authors addressed the questions, comments and suggested revisions proposed by the reviewers. I have no further comments or suggestions.

Reviewer #3

(Remarks to the Author)
I am completely satisfied with the revised manuscript.

Reviewer #4

(Remarks to the Author)
The authors have provided comprehensive answers to the questions posed to them, and I therefore consider the manuscript ready for publication.

Open Access This Peer Review File is licensed under a Creative Commons Attribution 4.0 International License, which permits use, sharing, adaptation, distribution and reproduction in any medium or format, as long as you give appropriate credit to the original author(s) and the source, provide a link to the Creative Commons license, and indicate if changes were made.
In cases where reviewers are anonymous, credit should be given to 'Anonymous Referee' and the source.

The images or other third party material in this Peer Review File are included in the article's Creative Commons license, unless indicated otherwise in a credit line to the material. If material is not included in the article's Creative Commons license and your intended use is not permitted by statutory regulation or exceeds the permitted use, you will need to obtain permission directly from the copyright holder.
To view a copy of this license, visit https://creativecommons.org/licenses/by/4.0/

We thank the reviewers for their critical comments and positive assessment of our work. Our responses are highlighted in blue text.

Reviewer #1 (Remarks to the Author)

STX is a paralytic toxin that inhibits Nav activity, blocking bioelectrical signaling. Given that STX can be deadly and poses a health risk to humans, there is interest in developing an effective antidote. Sxphs are STX binding proteins that provide STX resistance to frogs. In this manuscript, Nixon et al. test the hypothesis that Sxphs act as STX sponges that can reduce neurotoxicity and lethality in mice. Nixon et al. combine in vitro and in vivo experiments to show that Sxphs reduce neurotoxic effects and lethality in mice.

There is a substantial body of literature on STX and Sxphs, and this study builds on previous work by testing "toxin sponge" countermeasure hypotheses using multiple paradigms in a mouse model. The strength of this study lies in the combination of in vitro and in vivo experiments (including three paradigms to assess the efficacy of Sxphs to counteract STX neurotoxicity in mice) showing the capacity of Sxphs to reduce the effects of STX on both Nav targets and mice (both neurotoxic effects and lethality). The proteomic data showing the presence of Sxphs in different organs after i.p. injections is also a strength of the paper. The findings support the idea that Sxphs can be used to develop an antidote to STX. The potential to use Sxphs to develop STX antidotes could have significant impacts on human health and food safety.

The experimental design, data, and presentation are solid, and the statistical tests are appropriate. Overall, the data support the author's interpretations and conclusions. In addition, the text is written in a clear and concise manner, and the literature is appropriate. There are a few typos that should be revised, otherwise there are no suggestions for substantial revisions.

We thank the reviewer for their positive assessment of our work. We have addressed the typos in the revision.

Reviewer #2 (Remarks to the Author)

Dear Authors,

I had the pleasure of reading and reviewing the submitted article titled: "Saxiphilin functions as a 'toxin sponge' protein that counteracts the effects of saxitoxin poisoning". The authors have an excellent voice and convey the topics with clarity and a depth of understanding that makes the manuscript easy to digest. The article discusses their continued work with "toxin sponges", primarily Sxph, as a mediator for neurotoxin STX. What is new in this article compared to their past work is primarily the inclusion of animal models susceptible to the neurotoxin's deadly effects and the protection and recovery that Sxph offers. I will not be commenting on the multitude of very well described technologies such as electrophysiology and will be focusing on the proteomics experimentation and results. While the initial results are promising, there is a lack of detail in the description of the sample preparation, mass spectrometry data acquisition and the mass spectrometry data analysis. Without additional details, the results are unable to be evaluated correctly

or with a standard desired by the field. The experiments themselves could be sound, but without more information, making those judgements is challenging. Most noteworthy is the claim that Sxph is present in all harvested organs treated but not in those untreated. More interpretation of the results is required to validate with high confidence. Supplemental table 5, for example, utilizes software-specific headers which would be best described for those either without software access and/or in a vendor-neutral manner. With better header descriptions, the raw data comparisons may make more sense and agree with the authors' claims. Secondarily, the proteomics results were utilized, though only briefly mentioned, to validate safety. The authors do not mention what parameters were used to evaluate the proteome in this manner. I have broken down my specific thoughts and comments below and by section so that hopefully the authors can remark on these concerns in a manner this is expedient to them.

Intro

"Electrophysiological experiments have shown that binding of STX and STX congeners to this site endows Sxphs with the ability to rescue NaVs from block by these toxins." This sentence reads awkwardly and the narrative would be more easily followed if this were improved. "…and that the high-affinity of Sxph:STX interaction is stably maintained across a wide temperature range that includes typical mammalian body temperatures."

Rewritten as follows (p. 3): Electrophysiological experiments have shown that STX and STX congener binding to this site endows Sxphs with the ability to rescue Na_Vs from block by these toxins[26,30].

And on p. 4

Using *in vitro* assays, we find that the prototypical 91 kDa Sxph from the American bullfrog (*Rana catesbeiana*) (*Rc*Sxph)[11,26,27] rescues human Na_V channels from STX block, and that the high-affinity nature of the Sxph:STX interaction persists across a wide temperature range that includes typical mammalian body temperatures.

Can the authors please explain why the interaction between protein and toxin would be affected by various temperature regimes or provide a reference for the readers to indicate the relationship between temperature and interaction?

This point is addressed on p. 6 and the reference for prior studies of the temperature dependence of Sxph-STX interactions is included (ref. 29). Because all reactions are temperature dependent, we wanted to be sure that the affinity of Sxph for STX was not reduced at mouse temperature (~36°C) relative to what had been previously measured and room temperature or lower, as such a situation could negatively influence the efficacy of Sxph. We include a key reference that highlights the effects of temperature on binding reactions as requested (ref. 34). The rationale is rewritten as follows (new text in italics):

Studies of STX binding to *Rc*Sxph at ambient temperature (23 ± 2°C)[26,29,30] or lower (0 to 25°C)[29] show that STX affinity is not strongly perturbed over this temperature range. Before

embarking on experiments to test the effectiveness of *Rc*Sxph as an STX countermeasure in mice where the body temperature is ~36°C[33], we wanted to examine the temperature dependence of STX binding at temperatures >25°C to understand if the elevated temperature would compromise interaction with the toxin, *as temperature is a key factor affecting all binding reactions*[34].

Protein digestion

- Part numbers would be appreciated for the reagents used in this protocol, particularly the model number of the BCA assay kit and model numbers for the lysis buffer solutions so those attempting to follow the method can be assured they are selecting the correct purity.

We have added the following details as suggested:
BCA assay kit: BCA1-1KT Bicinchoninic Acid Kit for Protein Determination - Sigma-Aldrich p. 27.
Tris buffer: T1503 Trizma® base - Sigma-Aldrich p. 27
SDC: 30970 Sodium deoxycholate - Sigma-Aldrich p. 27
TCEP: 20491 TCEP HCl - Sigma-Aldrich p. 27
CAA: 22790 – 2-Chloroacetamide - Sigma-Aldrich p. 27

- I do not see the additional of any reagents that would be used for reduction and alkylation of the proteins prior to digestion (typically IAA and DTT. Were these added? Do you anticipate that not modifying these residues would affect your results? If you did reduce and alkylate, please state where in the protocol and how this was performed.

Reduction and alkylation were performed using Tris(2-carboxyethyl)phosphine (TCEP) and chloroacetamide, respectively. This is described in the Materials and Methods section (p. 27).

- What manufacturer or kit were you using for the SP3 beads? - A figure or a more detailed protocol for the steps used by the automated sample handler would be appreciated as it will help the reader follow the steps utilized even without the specialized lab equipment.

We have added the following details as suggested (p. 28):
Merck SpeedBeads magnetic carboxylate modified particles GE65152105050250 and GE45152105050250

Details for the steps used in the automated sample handler are as follows (p. 28):

Briefly, 65 µL of sample was added to 65 µL of 100% ethanol and mixed with the SP3 beads. After binding, the beads were washed three times with 80% ethanol. After washing, samples were digested in 50 mM TEAB at 40°C with 1 µg of trypsin (Thermo Scientific, Mass spectrometry grade Trypsin 90057) for two hours, then another 1 µg of trypsin was added and digested overnight at 37°C. After digestion, samples were acidified with trifluoroacetic acid

(TFA, final concentration 1% (v/v)) and peptides were desalted using in-house made stage tips packed with C18 disks (Empore) as described[56].

- Was the overnight digestion also performed at 40°C?

The overnight digestion was performed at 37°C. We have now included this on p. 28

- Please describe the trypsin used for these experiments by manufacturer, catalogue number, etc. Was the trypsin used MS-grade?

We have added the following detail as suggested (p. 28):
Thermo Fisher Mass Spectrometry Grade Trypsin: 90057 - Thermo Scientific

nLC-MS/MS
- While not entirely necessary, providing a part number for the column used would be beneficial to readers or those trying to repeat results.

We have added the following detail as suggested (p. 28):
Thermo Scientifc ES903

- Was the column heated? If so, to what temperature?

The column was heated to 50°C. We now include this information on p. 28.

- Was the trap column heated? If so, to what temperature?

The trap column was heated to 40°C. We now include this information on p. 28.

- Manufacturers and grade of solvents used for LC-MS/MS should also be included here, especially if the formic acid was pre-mixed.

We have added the following information as suggested on p. 28.
LC-MS Grade Acetonitrile J.T.Baker - JB9821.2500GL
LC-MS Grade Water: J.T.Baker - JB9825.1000GL
LC-MS Grade Formic Acid: LiChropur - 5.33002.0050 - Merck

- Please include the model of the nano-LC/UHPLC used here.

We have added the following information as suggested on p. 28.
Thermo Scientific UltiMate 3000 RSLCnano UHPLC System.

- A full LC gradient would be appreciated, though I acknowledge that the peptide elution range was 60 minutes from 4 to 35% increasing in %B.

We now detail the full gradient as follows on p. 28 as requested.

Peptides were eluted with mobile phase B gradient as follows: 0-1 min isocratic 4% B, 1-6 min to 6% B, 6-55 min to 25%B, 55-64 min to 35%B, 64-65 min to 75% B.

- The peptides were converted to gas phase via nano electrospray ionization, which while still a form of desolvation, should be clarified. Including the model of ion source, considering Thermo's wide range, would be useful to the reader if attempting to repeat results.

We have added the following information as suggested (p. 28):
Thermo Scientific™ EASY-Spray™ Source ES081

- 4 x 10^5, the 5 should be superscript and or could be written as 4x10E5 for the ion count target.

Corrected

- Please ensure that m/z is italicized following normal conventions in mass spectrometry.

Corrected

- Carryover between injections can be a significant problem in LC-MS/MS; please describe the sample injection table including when blanks were injected and what types of blanks were utilized. Was any blank subtraction performed in analysis? While not entirely necessary, ensuring that blanks contain few proteins is crucial

A blank run using pure solvent A was injected between individual organs and experimental groups. Blank subtraction was not performed, as it is not routinely applied in label-free proteomics. Blank samples lack biologically relevant signals, and subtraction may distort quantitative information.

- What were the injection volumes? How much sample was injected onto column?

Injection volume was 4 μL, ~ 1 μg of protein. This information is now noted on p. 28.

- A table of instrument parameters would be useful in the supplemental including what was already provided and additionally spray voltage, ion transfer tube temperature, RF lens percentage or Vpp, injection time values, data type (centroid/profile), and other relevant MS-related parameters.

We have added the following details as suggested in a 'Methods' section in the Supplementary Material p. 13:

Spray voltage was set to 1600 V and ion transfer tube temperature to 275°C. MS1 scans of peptide precursors were analyzed in Orbitrap in range 145-1450 *m/z* at 60K resolution and with following settings: RF Lens 60%, maximum injection time 123 ms, AGC target 100. DIA scans were performed in Orbitrap at 30K resolution. AGC target was set to 1000 and maximum injection time mode to Auto. Precursor mass range 400-1000 *m/z* was covered by 30 windows 20 Da wide. Activation type was set to HCD with 28% collision energy.

Proteomic Data Analysis
- Inclusion of the FASTA file into MASSIVE or another repository would be ideal.

We have already provided GenBank entry U05246.1 in the MASSIVE deposit. This entry includes both the nucleotide and amino acid sequences of *Rc*Sxph. The reference mouse proteome is available from UniProt (UP000000589).

- An example of tables and values that are useful for data analysis can be seen here: https://pubs.acs.org/doi/full/10.1021/acs.jproteome.3c00671 this publication often serves as a good baseline for variables utilized within the Spectronaut software to ensure robust results. The authors did include more stringent search values than the default, which is greatly appreciated.

As noted by the reviewer, we applied more stringent search values than the default.

- How were the different organs searched? As a group? Separately? Spectronaut can often infer peptide identifications based on other datasets included in a group, so knowing the grouping can help the reader evaluate uniqueness between organ systems.

Both separate and concatenated searches were performed for the different organs, and no significant differences in Sxph content were observed between these two approaches.

- The authors state that they utilize Perseus for downstream analysis but do not provide a table or instructions on how to repeat their results (or even what Perseus was used for).

Perseus was used for proteomic data analysis. We have provided the additional text below to clarify (p. 29).

For samples where protein was not detected, missing signal values were imputed in Perseus[58]. Intensity values were Log2 transformed to achieve a normal distribution. To account for the limit of detection, missing values were optionally imputed from a normal distribution (width 0.3, downshift 1.8) using the software default settings. For the identification of differentially abundant proteins, a two-sample Student's t-test was applied. Statistical significance was defined using a permutation-based False Discovery Rate (FDR) of 5% (0.05) and a minimal fold-change parameter S0=0.1.

Results

- Non-endogenous proteins can often undergo modification when introduced into a host system. Did the measured amount of Sxph in the organ systems track how much Sxph was introduced to the animal? Was there an expected loss of intact protein due to modifications?

> No modifications were detected. While we currently have a single 5-hour time point, the observed intensity of Sxph is consistent with systemic exposure. Given the disulfide architecture (21 disulfides) of the protein, and high thermostability of Sxph, it is unlikely that Sxph is significantly modified.

- How much Sxph was detected in organ system blanks? Theoretically, there should be none, but this would be excellent confirmation of LC-MS/MS related systematic carryover between injections and/or confirmation that the data analysis pipeline did not introduce any false positives downstream.

> As noted on p. 14, no Saxiphilin was detected in organ system blanks.

- By what definition is a “stress-response” protein? This claim would be better supported by a pathway analysis utilizing the quantitative nature of the data (as described in the methodology).

> Stress-response protein yeswas defined using standard gene ontology (GO) annotations for proteins involved in cellular stress-related pathways. https://www.ebi.ac.uk/QuickGO/term/GO:0006950 .
>
> To clarify, we added the following to the description of Table S5 in the supplementary material:
>
> Stress proteins were identified using standard gene ontology (GO) annotations https://www.ebi.ac.uk/QuickGO/term/GO:0006950.

- A cursory inspection of the proteomics data does yield a concerning result which would be alleviated with a deeper description of the injection organization and search results. Uromodulin, a protein exclusively secreted by the kidney in the thick ascending limb, was present in the liver proteomics supplemental data table. Providing definitions for the headers for each column (for example, does NrOfStrippedSequencesIdentified mean, in layman terms, number of unique tryptic peptides?).

> Uromodulin was consistently and correctly detected in all five kidney samples. Among the remaining 19 samples from other tissues, this protein was identified in only one liver sample at roughly tenfold lower abundance than in the kidney samples.

The signal observed in the liver most likely represents a false positive. This has no impact on the data interpretation or overall conclusions of this study.
As requested, we modified the headers in Table S5 to clarify the column contents.

- Is there an experimental reason why the liver and skeletal muscle have ~2000 fewer identified protein groups as the other organs? Did these samples have less on-column load?

Column load was consistent across samples. In our dataset, the lowest number of protein identifications was observed in the gastrocnemius skeletal muscle sample (4617 proteins). This is a known technical limitation in proteomic analyses, primarily due to the high abundance of structural and contractile ("ballast") proteins that are intrinsic to muscle tissue and can suppress detection of lower-abundance proteins. The number of proteins identified in liver (5248 proteins) is comparable to heart (5703 proteins), with ~2000 more proteins being detected in kidney (7323 proteins) and brain (7707 proteins). We note that these values are aligned with other mouse organs proteome experiments, such the comprehensive mouse survey by Luo *et al.* (PMID: 36058520) who report organ protein identifications as follows: gastrocnemius muscle, 3263; liver, 5108; heart, 5335; kidney, 6911; and brain, 6966; suggesting no experimental anomaly in our work.

- Can the authors comment on how bioavailability in multiple organ systems can be beneficial for a sodium-channel blocking neurotoxin like STX?

Na_Vs are present in essentially all organs. STX has been shown to distribute widely and can cross the blood-brain barrier. Systemic bioavailability of Sxph across multiple organs is advantageous for neutralizing a systemically distributed sodium channel blocking toxin such as STX. We now note this point in the discussion (p. 17) as follows:

> Because STX can reach many organs and can cross the blood-brain barrier[46], the ability of Sxph to follow a similar distribution pattern may contribute to its effectiveness as a toxin sponge.

We have no information on why it is beneficial for STX to be able to reach so many different organs. STX is an environmental toxin made by algae and cyanobacteria and is not used by organisms for defense or predation. Thus, it is not possible to speculate on a rationale for why this molecule is such a potent toxin. To the best of our understanding, the biological function of STX in the marine and fresh water organisms that produce this toxin remains unknown. It is certainly a very interesting question as to why single-celled organisms produce a toxin that is so potent against the nervous system of multicellular organisms. As far as we know, there are no answers, or even credible speculation, regarding this intriguing relationship.

- Where in the supplemental table 5 does it indicate that there was no Sxph signal? The binary results are generally some sort of computation related back to either integrated intensity from the chromogram for each ID'ed peptide in the protein group. So, in the kidney data table, row 7326 for Sxph, 40 peptides were able to be mapped across all samples in the group (assuming just kidney) but the vehicle, while in lower quantity, did have measurable signal for this protein. If there is additional computation occurring for this table in which the magnitude of difference between the vehicle and dosed samples shows some statistical significance, please describe.

> Saxiphilin was not detected in any organ from vehicle mice in our proteomics experiments. Hence, all intensity values for this protein were missing. To compare fold changes in protein abundance between vehicle and Sxph treated mice (Fig.5), we used the impute missing values function in Perseus, such that the missing values were replaced by random numbers drawn from a normal distribution of 1.8 standard deviation down shift and with a width of 0.3 for each sample, simulating low abundance proteins (Tyanova et al., *Nature methods* 2016).
>
> To make this point clear, we have now edited Supplementary Table 5 to include the original values alongside the imputed values. Samples where a protein was not detected are marked with 'NaN'.
>
> We have further clarified this in the methods as follows (p. 29):
>
> Fold-changes in protein abundance between vehicle and Sxph-treated mice were examined by comparing mean signal intensity. For samples where protein was not detected, missing signal values were imputed in Perseus[59].

- Was supplemental figure 5 generated with a combination of all ID'ed peptides for the protein across all organs? Why not just perform LC-MS/MS on the isolated protein to perform this experiment? Was the binding pocket region mapped by LC-MS/MS? That would be an interesting result to highlight, particularly if the pi-interactions are crucial for the binding affinity.

> Yes, this figure shows the shared peptides across all organs as stated in the figure legend. All samples show coverage across the C-lobe that contains the toxin binding pocket. This toxin binding site and has been extensively characterized using X-ray crystallography and binding assays in our previous work (Refs. 26, 27, & 30). The figure shows that we detect peptides spanning each domain of the Sxph sequence in samples recovered from the various organs. We think this is important as it demonstrates that we cover the whole protein rather than fragments from a few Sxph domains.

I appreciate the immense work that has gone into this study and hope that my concerns are alleviated with additional experimental details.

We thank the reviewer for the detailed evaluation of our work. We hope that we have answered their concerns.

Reviewer #3 (Remarks to the Author)

In this article, Nixon and her colleagues reexamine the ability of saxiphilin (Sxph), a protein produced naturally by certain species of frogs, to sequester saxitoxin (STX), a highly potent neurotoxin generated by microalgae that can accumulate in shellfish. By blocking voltage-gated sodium channels, STX triggers severe symptoms within minutes potentially leading to death and requiring urgent medical intervention. Using in vitro tests and mouse models, the authors demonstrate that RcSxph from the American bullfrog effectively neutralizes STX on several subtypes of human sodium channels and functions effectively as a “toxin sponge.” In mice, a single intraperitoneal dose of RcSxph protected against lethal exposure to STX, whether administered before, during, or after toxin exposure. This protection was dependent on an intact STX binding pocket, confirming direct sequestration of the toxin in vivo. RcSxph was shown to rapidly distributed throughout the body, allowing it to capture circulating STX and prevent neurotoxicity. Compared to previous antiserum-based strategies, RcSxph was more effective at higher toxin doses without requiring intravenous administration or encapsulation. The protein was well tolerated, stable, and non-toxic. It should be noted that Sxphs bind to several STX congeners, suggesting broad protection against these toxins. Overall, these results validate the “toxin sponge” hypothesis and highlight Sxphs as promising and scalable antidote candidates for STXs and related neurotoxins.

As with the group's previous work, the experiments are exceptionally well conducted and the results are supported by rigorous and appropriate controls. The stated goal of developing a new strategy to counter saxitoxin poisoning in mammals, as well as its experimental validation, are clearly and convincingly demonstrated. That said, several aspects of the discussion could be further developed to strengthen the broader interpretation of the results. The authors explore three different approaches to Sxph administration—before, during, or after STX injection—representing three distinct paradigms that all result in effective toxin neutralization. While these results highlight the potential of Sxph as a preventive, protective or therapeutic agent, and provide valuable insights into its mechanism of action (particularly with regard to its ability to cross the blood-brain barrier), additional context would help clarify their relevance in real-world scenarios of food poisoning.

For example, the practical value of administering an antidote preventively, or even at the time of ingestion, may be limited, since it would generally suffice to avoid consuming contaminated shellfish.

Pre-administering Sxph mimicks the natural scenario where frogs endogenously express this protein and enables us to test the toxin sponge hypothesis model of toxin resistance. To highlight this point, we now include the following on p. 9 of the ‘Results’ (new text in italics):

Together, these data demonstrate that prophylactic i.p. administration of Sxph is an effective way to counter the lethal effects of STX *and support the idea that the natural function of Sxphs in frogs is to protect from toxin exposure*[6,11].

And in the 'Discussion' on p. 15-16 as follows (new text in italics):

These findings confirm that Sxphs use this pocket to sequester the toxin *in vivo,* matching results from *in vitro* binding and functional studies[6,26,30], clearly establish that the ability of *Rc*Sxph to counter STX depends on its high affinity STX binding site, *and support the idea that Sxphs in frogs provide protection from toxins*[6,11].

We agree, no one is going to take the antidote in order to consume contaminated shellfish.

Furthermore, as both the toxin and the antidote were administered intraperitoneally, an oral exposure model might better reflect the natural route of poisoning if the aim is to propose a strategy applicable to the ingestion of contaminated food. With this in mind, the results of a previous study, rightly cited in the manuscript, could have been discussed (Finch et al., 2023). It would also be interesting to test sublethal doses of STX and inject RcSxph after the first symptoms appear.

We aimed to test the toxin sponge hypothesis and assess whether Sxphs can counteract the lethal effects of STX. Our data show that Sxph was effective at neutralizing a lethal dose of the toxin directly administered by injection. Given that Sxph can counter STX in this maximalist situation, we do not think that Sxph would be less effective on lower toxin doses or in situations where the toxin is ingested. Demonstrating this point would require a different study that would be interesting, as the reviewer notes. Hence, we did not comment on its use for sub-lethal exposure. We now mention the importance of testing antidotes in ingestion paradigms as suggested as follows (p. 19):

Advancing Sxph inspired antidotes will require efforts to enhance affinity, ensure effectiveness against a range of PSTs, address any potential immunological responses, optimize delivery routes, and show that they are effective in a variety of exposure scenarios including toxin ingestion[35].

Finally, the immunogenicity of RcSxph, a 91 kDa protein, could pose a significant problem in the event of repeated use as an antidote. This point could also be discussed and solutions proposed.

We are not aware of reports of repeated STX poisonings. Hence, we expect that if Sxph were used as a treatment, it would be administered once, reducing concern

regarding immunogenic effects related to repeated administration. For this reason, we did not elaborate on the possibility of repeated use as this is an unlikely scenario.

There are many factors that need to be addressed to optimize this protein, or derivative thereof, as an STX antidote, even for a single use. Such modifications could introduce new immunological issues that are not apparent with unaltered Sxphs. To highlight this point further and address the general concern about immunogenicity, we added the following to the final paragraph of the 'Discussion' (p. 19) (new text in italics)

Our results demonstrate that it is possible to harness the American bullfrog *Rc*Sxph as an STX countermeasure and that administration of Sxph alone is sufficient to rescue and protect against the neurotoxic and lethal effects of STX poisoning, validating the idea that Sxphs act as 'toxin sponges' *in vivo*[6]. *Advancing Sxph inspired antidotes will require efforts to enhance affinity, ensure effectiveness against a range of PSTs, address any potential immunological responses, optimize delivery routes, and show that they are effective in a variety of exposure scenarios including toxin ingestion*[35]. The diversity of the Sxph family[26,30] provides a scaffold for development of variants having enhanced affinity and broad specificity for PSTs and points to the potential of capitalizing on the natural array of anti-toxin proteins[28,31,41,44,48,49] as blueprints for developing toxin antidotes.

Reviewer #4 (Remarks to the Author):

Using mouse models of STX poisoning, Nixon and co-workers demonstrated that Saxiphilins (Sxphs) have the potential to be an effective anti-STX agent. Indeed, they showed that RcSxph from the American bullfrog (Rana catesbeiana) efficiently counteracts the lethal effects of STX poisoning in vivo, in three different murine toxin challenge paradigms, each of them involving injection in mice of a STX dose known to induce the rapid onset of paralysis, seizures, convulsions, and death within few minutes. RcSxph rapidly distributes in mice organs, including brain, and it appears to be tolerated in mice without any apparent acute toxicity or adverse reactions, at least for the entire duration of the experiments. I found the work highly compelling in defining Sxphs as a prototypical antidote for the lethal toxin STX, and more in general, for paralytic shellfish toxins, of which STX is the archetype. In this regard, I found it particularly strickling that a single dose of RcSxph counteracts STX toxicity/lethality in mice in either order of STX and Sxph administration (together or sequentially).

Also, the preliminary biochemical characterization of RcSxph stability/binding affinity for STX (i.e., ITC experiments) is clear and thus convincing. I just have a curiosity:

The authors reported that RcSxph has an apparent melting temperature (Tm) of ~55°C. I wonder whether the authors encountered 'heat issue' in the baseline stabilization of ITC measurements performed at 50°C. This is, indeed, a value close to the reported melting temperature of the protein and, therefore, folding and unfolding phenomenon should equally occur in this condition. Did the authors see the occurrence of this thermodynamic

folding and unfolding equilibrium, perhaps visualized in a longer time to reach baseline stabilization and/or in a significantly different heat value of the baseline with respect to ITC measurements performed at 25°C and 35°C?

We did not observe any temperature-related issues during data acquisition. The time required for baseline stabilization, the return-to-baseline after injections, and peak shapes were comparable at 25 °C, 35 °C, and 50 °C. The nearly temperature-independent ΔG values (–12.3 to –12.0 kcal mol^{-1}) and the preserved stoichiometry (N ≈ 1 at all temperatures) indicates that there are no significant contributions from a folding-unfolding equilibrium under these conditions. We note that the TF assay is an indirect measure of folding, and while it is useful as an initial screen for toxin-protein interactions, we do not know how accurately it reflects the actual unfolding reaction.
